# DL-GapFilling: a novel deep learning framework for improved plant genome gap filling

**DOI:** 10.1093/bib/bbag007

**Published:** 2026-01-26

**Authors:** Yu Chen, Zihao Wang, Gang Wang, Guohua Wang

**Affiliations:** College of Computer and Control Engineering, Northeast Forestry University, Harbin 150040, China; College of Computer and Control Engineering, Northeast Forestry University, Harbin 150040, China; College of Computer and Control Engineering, Northeast Forestry University, Harbin 150040, China; College of Computer and Control Engineering, Northeast Forestry University, Harbin 150040, China; Department of Computer Science and Technology, Faculty of Computing, Harbin Institute of Technology, Harbin 150001, China

**Keywords:** genomes, assemblies, gaps, DL, GapFilling

## Abstract

Genome assembly has been a cornerstone of bioinformatics for decades, with faster and more accurate assembly of unknown genomes remaining a critical challenge. However, genome diversity, structural variations, insufficient sequencing depth, and limitations of current algorithms often lead to numerous gaps during assembly, hindering the construction of high-quality reference genomes. While various assembly methods and software tools have been developed, most exhibit low efficiency in gap filling and fail to account for the intrinsic structural properties of genomic sequences. Here, we present DL-GapFilling, a deep learning-based framework for genome assembly and gap filling. DL-GapFilling leverages a novel Deep Filling Neural Network model to efficiently extract and contextualize flanking sequence information, and incorporates the BeamStar contraction-expand algorithm, which integrates a redefined cost function, an enhanced search strategy, and genomic structural priors to improve both generalization and efficiency in gap filling. In addition, a PredictionFilter mechanism is introduced to selectively retain high-confidence predictions, mitigating the impact of poorly predicted sequences on assembly quality. Experimental results demonstrate that DL-GapFilling significantly improves gap-filling performance across multiple plant or algal genome datasets, achieving increases of 15.6%, 6.1%, 16.7%, 5.5%, and 23.5% in the number of gaps filled compared to traditional tools, and outperforming existing DL-based methods in both efficiency and accuracy. These findings underscore the potential of DL-GapFilling as a powerful tool for advancing genome assembly research.

## Introduction

The popularization of second-generation sequencing technology has made genome sequencing more efficient, and has promoted the determination of reference genomes for more species. However, the insufficient sequencing depth, repetitive sequence structure, and technical limitations of second-generation sequencing often result in unidentified gaps in genome assembly. Filling gaps usually requires additional sequencing data or computational prediction methods. The main methods currently include multi-software assembly, the use of reference genomes from related species, polymerase chain reaction amplification of end gaps, and improved assembly methods based on De Bruijn graph [1].

In the field of genome assembly, many tools have been developed to meet the needs of different sequencing technologies. For second-generation sequencing (short-read length) data, tools such as Velvet [2], ABySS2 [3], SPAdes [4], IDBA-UD [5], ALLPATHS [6], ALLPATHS-LG [7], SOAPdenovo2 [8], SKESA [9], MEGAHIT [10], and metaMDBG [11] focus on processing high-throughput short-read-length data, which are able to effectively deal with the problems of gaps and duplicate regions that may be encountered by the short-read-lengths in the assembly process. These tools utilize the high coverage of short-read length data to provide high-quality assembly results. In addition, to further enhance the quality of assembly, specialized gap filling tools such as HaploMerger2 [12], Sealer [13], GapFiller [14], TIGRA [15], Pilon [16], and FGAP [17] have been designed to fill the gaps during the assembly process and optimize genome contiguity by leveraging the paired-end information and local coherence of short-read lengths. However, the reliance of second-generation sequencing technologies on assembling short fragments to construct genome sequences imposes significant limitations, particularly in resolving repetitive sequences and structural variants within complex genomes. This underscores an urgent need for advancements in sequencing technologies capable of producing longer read lengths.

With the emergence of third-generation sequencing technologies (PacBio SMRT and Oxford Nanopore), long-read length assembly tools such as Canu [18], HiCanu [19], Flye [20], RFfiller [21], Wtdbg2 [22], Shasta [23], NextDenovo [24], and Verkko2 [25] were developed for this application, and the gap tools for its assembly are TGS-GapCloser [26], Racon [27], and LR GapCloser [28]. These tools are good at handling long-read length data and are able to span complex repetitive regions, which significantly improves the integrity and continuity of the genome. In terms of improving base-level accuracy, tools such as NextPolish [29], NextPolish2 [30], PEPPER-Margin-DeepVariant [31], DeepConsensus [32], Apollo [33], and DeepPolisher [34] can perform multiple rounds of polishing on the assembled sequence, thereby optimizing the sequence quality near gaps. In order to better combine the advantages of short- and long-read length data, hybrid assembly tools such as HybridSPAdes [35], OPERA-LG [36], MaSuRCA [37], Unicycler [38], HASLR [39], SAMBA [40], and DENTIST [41] were developed. These tools provide a more reliable basis for genome assembly by integrating different types of sequencing data and maximizing the high accuracy of short-read lengths and the coverage advantage of long-read lengths. In recent years, deep learning has made great progress in predicting unknown regions and repairing sequence deletions, which offers new possibilities for gap filling. For example, Multivariate Variational Mode Decomposition (MVMD) [42] extracts features from multi-channel electroencephalography (EEG) and combines it with long short-term memory (LSTM) for high-precision classification, demonstrating the potential of multivariate signal decomposition and deep learning in complex pattern recognition. In the post-processing stage of genome assembly, deep learning tools have also been increasingly applied: NextPolish [29], NextPolish2 [30], DeepConsensus [32], Apollo [33], and DeepPolisher [34] are used for error correction; DeepTrio [43] enables haplotype phasing; and DeepMicrobes [44] can identify and remove contaminated sequences. At the same time, the integrated hybrid assembly and post-processing tool Verkko2 [25] can achieve end-to-end optimization for assembly, gap filling, and phasing, improving the continuity and accuracy of complex eukaryotic genomes. Gappredict [45] uses LSTM based on the sequence information flanking the gap to predict and fill missing sequences, improving assembly quality, while DLGapCloser [46] integrates homologous genome mapping to optimize gap sequence prediction.

All of them can improve the assembly quality by expanding the assembly sequences to fill the gap. However, there are problems such as the prediction network structure involving only a single layer, which is too simple, and the sequence prediction algorithm only considers the probability value of each base. We propose a novel method, DL-GapFilling, which introduces three key innovations: (i) Deep Filling Neural Network (DFillingNet) for efficient extraction of flanking sequence information; (ii) the BeamStar Shrink-Expand Algorithm (BSCEA) for integrating cost functions and optimizing sequence predictions, enhancing gap-filling generalization and efficiency; and (iii) the PredictionFilter mechanism, which retains high-confidence predictions and mitigates the effect of low-quality sequences on assembly. Evaluation on multiple datasets demonstrates DL-GapFilling’s superior performance and robustness.

## Materials and methods

**Figure 1:**
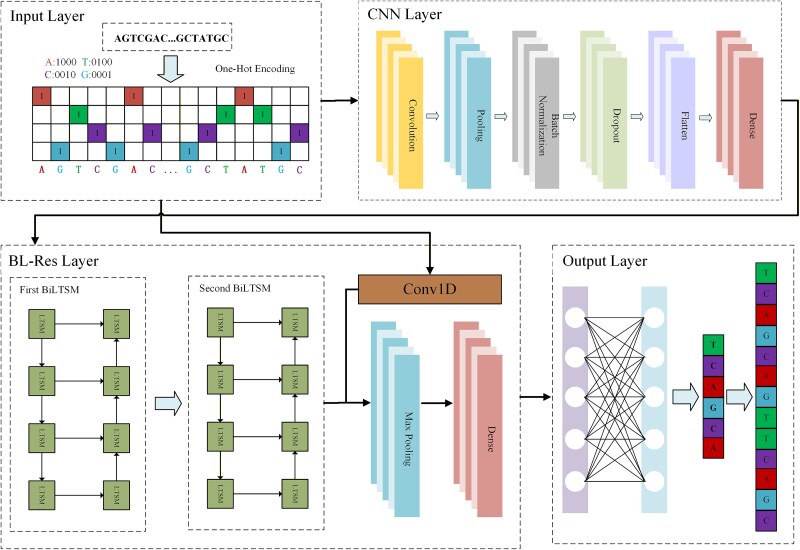
DFillingNet network architecture for gene sequence prediction; DFillingNet comprising four key components: the input layer, the CNN layer, the BL-Res layer, and the output layer.

Gaps in gene assemblies are often caused by sequencing errors, repetitive sequences, insertions, deletions and under-coverage, and this problem is further exacerbated by the limitations of the assembly algorithm. To address this problem, deep learning is employed to predict gap sequences by analyzing the flanking regions surrounding the gaps. The DL-GapFilling method uses the DFillingNet model, which consists of an input layer, a Convolutional Neural Network (CNN) layer, a BL-Res layer, and an output layer. The specific flowchart is shown in Fig. 1. The input layer encodes the DNA sequence in one-hot format to convert it into a high-dimensional vector. The CNN layer extracts local features, while the BL-Res layer employs stacked bidirectional long short-term memory (BiLSTM) units with residual connections to model long-range sequential dependencies. A one-dimensional CNN implements a residual connection between the BiLSTM layers to alleviate the gradient propagation problem. In the sequence expansion stage, the BeamStar contraction-expansion algorithm (BSCEA) is employed, which is an effective search algorithm for optimizing the expansion of the predicted sequence. In addition, a screening mechanism, PredictionFilter (Fig. 3), is proposed to effectively remove low-quality sequences generated during prediction, thereby improving the accuracy of gap filling.

We use one-hot encoding to convert each base into a unique binary vector. The base information contained in the flanks on both sides of the gap is represented by one-hot encoding, in which each base (A, T, C, G) is converted into a four-dimensional vector.

### Model architecture

DFillingNet consists of four components: the input layer, CNN layer, BL-Res layer, and output layer (Fig. 1). DNA sequences are encoded in one-hot format at the input layer, converting them into high-dimensional vectors. The CNN layer extracts local features and facilitates hierarchical learning, while max pooling, batch normalization (BN), and dropout enhance efficiency and prevent overfitting. The BL-Res layer captures bidirectional dependencies using stacked BiLSTM units and residual connections to alleviate the vanishing gradient problem. The output layer iteratively expands nodes to predict the sequence and fill gaps.

The CNN layer contains operations such as convolutional operations, local pooling, BN, dropout, etc. We use a convolution kernel size of 3 and an activation function of ReLU. In the input sequence $\mathrm{X}=\left\{{X}_1,{X}_2\dots, {X}_T\kern0em \right\},{X}_t\in{R}^{d_x\times T}$, the size of the convolution kernel is k, the number of convolution input channels is ${d}_h$, $L$ is the length of the input sequence, and the output sequence is $Y\mathbf{\in}{R}^{C_{out}\times{L}_{out}}$. For the ${C}_{out}$ th output channel at the $t$ th position, the convolution operation is given by:


(1) \begin{equation*} {y}_{c_{out},t}=\sum_{c_{in}=1}^{C_{in}}\sum_{k=1}^K{w}_{c_{out},{c}_{in},k}\cdotp{x}_{c_{in},t\cdotp S+k-P}+{b}_{c_{Out}} \end{equation*}

where ${w}_{c_{out},{c}_{in},k}$ is the weight of the convolution kernel, ${x}_{c_{in},t\cdotp S+k-P}$ is the value in the input sequence involved in the convolution, and ${b}_{c_{out}}$ is the bias. The convolution operation extracts local features of the input data through local feature extraction, parameter sharing, and translation invariance, while reducing the number of parameters, thus improving computational efficiency.

BN mitigates the gradient problem in deep networks by normalizing the activation values to a zero mean and unit variance. The convergence is accelerated by reducing the range of variation of activation values in different layers. Its formula is as follows:


(2) \begin{equation*} {y}_i=\gamma \bullet \frac{x_i-{\mu}_B}{\sqrt{\sigma_B^2+\epsilon }}+\beta \end{equation*}

where ${x}_i$ is the input eigenvalue, ${\mu}_B$ is the mean of the batch data, ${\mathrm{\sigma}}_B^2$ is the variance of the small batch data, $\gamma$ is the scaling parameter for learning, and $\beta$ is the learnable offset parameter.

The BL-Res layer consists of two BiLSTM layers and a maximum pooling layer, where each BiLSTM layer consists of forward and inverse LSTMs for capturing bi-directional contextual information of the sequence data. The output of the CNN layer is used as an input to the first BiLSTM layer, which is processed by the forward LSTMs and the inverse LSTMs to obtain a bi-directional feature representation. The formula is as follows:


(3) \begin{equation*} {h}_{BiLSTM1,t}=\left[{\overrightarrow{h}}_{BiLSTM1,t};{\overleftarrow{h}}_{BiLSTM1,t}\right] \end{equation*}

(4) \begin{equation*} {\overrightarrow{h}}_{\!\!BiLSTM1,t}={LSTM}_{fwd}\left({h}_{conv}\left[t\right],{\overrightarrow{h}}_{BiLSTM1,t-1}\right) \end{equation*}

(5) \begin{equation*} {\overleftarrow{h}}_{\!\!BiLSTM1,t}={LSTM}_{bwd}\left({h}_{conv}\left[t\right],{\overleftarrow{h}}_{BiLSTM1,t-1}\right) \end{equation*}

The second BiLSTM layer takes the output of the first layer as input and repeats the bidirectional processing to extract deeper bidirectional features. The corresponding formulas are given as follows:


(6) \begin{equation*} {h}_{BiLSTM2,t}=\left[{\overrightarrow{h}}_{BiLSTM2,t};{\overleftarrow{h}}_{BiLSTM2,t}\right] \end{equation*}

(7) \begin{equation*} {\overrightarrow{h}}_{\!\!BiLSTM2,t}={LSTM}_{fwd}\left({h}_{conv}\left[t\right],{\overrightarrow{h}}_{BiLSTM2,t-1}\right) \end{equation*}

(8) \begin{equation*} {\overleftarrow{h}}_{\!\!BiLSTM2,t}={LSTM}_{bwd}\left({h}_{conv}\left[t\right],{\overleftarrow{h}}_{BiLSTM2,t-1}\right) \end{equation*}

In order to solve the problem of gradient vanishing that may be caused by the deep network, this article introduces the residual connection [47], which adds the output of the second BiLSTM layer with the output of the one-dimensional convolutional layer. The output is obtained via residual addition, where the output from the second BiLSTM layer is combined with the output from the 1D convolutional layer. Here, ${h}_{conv}\left[t\right]$ denotes the feature vector at the ttt-th time step obtained from the convolutional layer. ${LSTM}_{fwd}\left(\cdotp \right)$ and ${LSTM}_{\mathrm{bwd}}\left(\cdotp \right)$ represent the forward and backward LSTM units, respectively. ${\overrightarrow{h}}_{\!\!BiLSTM1,t}$ and ${\overleftarrow{h}}_{\!\!BiLSTM1,t}$ denote the forward and backward hidden states of the first BiLSTM layer, and ${\overrightarrow{h}}_{\!\!BiLTSM2,t}$ and ${\overleftarrow{h}}_{\!\!BiLTSM2,t}$ denote those of the second BiLSTM layer.

Through the two BiLSTM layers, the model is able to obtain deep bi-directional feature representations for each time step, which are then used by the subsequent max-pooling layer to produce a fixed-length high-level feature vector. Finally, the output of the residual join is represented as:


(9) \begin{equation*} y\left[t\right]={h}_{BiLSTM2,t}+{h}_{conv1D,t} \end{equation*}

To further reduce computational complexity while preserving key features, the output after residual concatenation is processed through a maximum pooling layer using a sliding window.

The encoded gene sequence information will be output in the Output layer after being processed by the CNN layer and the BL-Res layer. This layer is mainly for the expansion of the expansion node. The seed sequence will keep growing until it expands into a predetermined length of predicted sequence. Here we construct a new method for predicting sequences (BSCEA) algorithm.

### BeamStar contraction–expansion algorithm

Beam search algorithms [48] are widely used for neural network decoding. Although their memory requirements are lower than those of breadth-first search, they may miss the global optimum. The A^*^ algorithm [49] combines path cost and heuristic evaluation, but it consumes a large amount of memory. Best-first beam search [50] improves the memory requirements by optimizing the scoring-based decoding strategy. Wave-beam search [46] adds the “contraction-expansion” pruning strategy to beam search, but it only considers probability values and may fail to find the global optimum. To address this problem, the BSCEA was developed, which incorporates additional influencing factors into the path evaluation process, optimizes path selection, improves algorithm performance, and enhances the quality of the results.

Currently, mainstream sequence prediction methods such as Hidden Markov Model (HMM) [51], Deep Learning (LSTM), or other machine learning methods [52] perform well when dealing with a large number of biological sequences, but the structural features of the sequences are usually neglected. To solve this problem, the BSCEA algorithm proposed in this article combines the A^*^ and BS algorithms in gene sequence prediction for the first time. BSCEA improves the search efficiency by dynamically adjusting the beam width: at the initial stage, the beam width is gradually relaxed to expand the search range; when it approaches the preset beam length, it starts to shrink to control the number of nodes and balance accuracy and resource consumption. At the end of the search, the algorithm selects the lowest cost among the candidate sequences as the final prediction result. Compared with the traditional BS and beam search, BSCEA retains more potentially superior solutions in the expansion phase to avoid early pruning, and accurately screens out the global optimal nodes in the contraction phase. As shown in Fig. 4, BSCEA integrates the multidimensional features of sequences to significantly optimize the performance of gene sequence prediction.

The cost function f(n) of the BSCEA algorithm comprises the actual cost g(n) and the heuristic cost h(n) [53]. The actual cost represents the cumulative uncertainty from the starting node to the current node n and is calculated as:


(10) \begin{equation*} newG= currentG+{\sum}_{i=1}^n\big| \mathit{\ln}\left({probability}_i\right)\big| \end{equation*}

where $currentG$ is the accumulated cost at the current node and ${probability}_i$ is the predicted probability of the iii-th base. The summation term quantifies the total prediction uncertainty along the path. The heuristic cost estimates the remaining cost to the target node and is evaluated as:


(11) \begin{equation*} Heuristic(S)=\alpha \cdotp H(S)+\beta \cdotp normRemLen+\gamma \cdotp CGRatioImpact \end{equation*}

Here, $H(S)$ is the sequence entropy, which quantifies sequence complexity [54]; normRemLen is the normalized logarithm of the remaining sequence length; and CGRatioImpact measures the guanine–cytosine (GC) content difference between the current sequence and the predicted sequence, reflecting biological plausibility [55]. The weighting coefficients $\alpha$, $\beta$, and γ balance these contributions. The GC content difference is defined as:


(12) \begin{equation*} \varDelta C{G}_{weighted}=\left|\frac{C_{next}+{G}_{next}}{\ {L}_{next}}-\frac{C_{current}+{G}_{current}}{L_{current}}\right| \end{equation*}

where ${C}_{current}$ and ${G}_{current}$ denote the counts of cytosine and guanine in the current sequence, ${C}_{next}$ and ${G}_{next}$ denote the corresponding counts in the next seed sequence, ${L}_{current}$ and ${L}_{next}$ are the lengths of the current and next sequences, respectively [55].

Integrating these components, the BSCEA heuristic function is formulated as:


(13) \begin{align*} Heuristic(S)=&-\alpha \cdotp \frac{\sum_{i=1}^np\left({x}_i\right){\mathit{\log}}_bp(x) dx}{maxEntropy}\notag\\&+\beta \cdotp \frac{\mathit{\ln}\left( totalLength- SeedLength\right)}{\mathit{\ln}(totalLength)}\notag\\&+\gamma \cdotp \left|\frac{C_{next}+{G}_{next}}{\ {L}_{next}}-\frac{C_{current}+{G}_{current}}{L_{current}}\right| \end{align*}

where $p\left({x}_i\right)$ is the predicted probability of the i-th base, $maxEntropy$ is the maximum possible entropy for a sequence of length nnn, $totalLength$ denotes the total predicted sequence length, and $SeedLength$ is the length of the current seed sequence. This formulation integrates sequence complexity, remaining length, and GC content disparity to guide the algorithm in selecting an optimal and biologically plausible expansion path.

### Overview of the DL-GapFilling method

**Figure 2:**
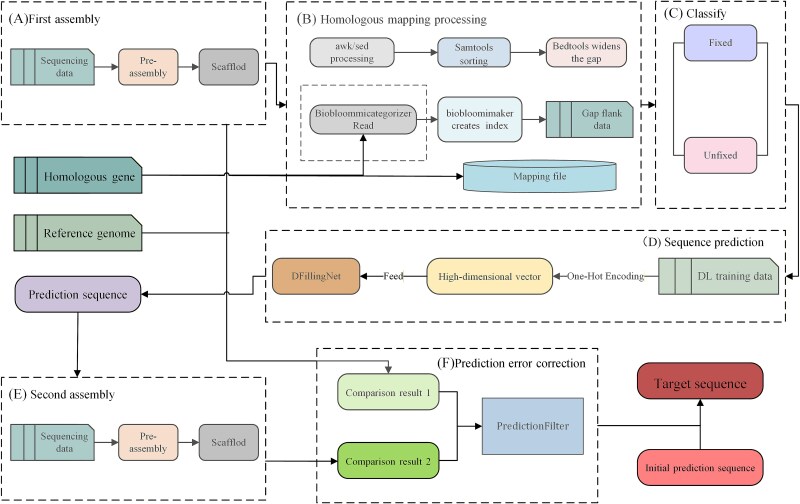
DL-GapFilling workflow overview.

Figure 2 schematically illustrates the DL-GapFilling workflow, which comprises three principal stages: (a) Data Preparation and Homology Mapping, (b) Gap Classification and Sequence Prediction, and (c) Hybrid Assembly and Error Correction.

Stage 1: Data Preparation and Homology Mapping. Short-read sequencing data are preassembled into a scaffold using conventional tools (Fig. 2a). This scaffold is subsequently processed to extract gap information using utilities such as awk and sed. To identify homologous regions, the gap sequences are sorted and expanded using alignment tools including SAMtools [56] and BEDtools [57]. Homologous gene mapping is then performed leveraging the HomoloGene database and tools such as OrthoMCL [58] and OrthoFinder [59]. Concurrently, read lengths are categorized, and mapping indices are constructed using BioBloomMICategorizer [60] and BioBloomMaker [60], respectively (Fig. 2b). This stage culminates in the precise identification of target gap sequences and their flanking regions.

Stage 2: Gap Classification and Sequence Prediction. The identified gaps are categorically divided into two groups: Fixed gaps, which are amenable to resolution by traditional gap-filling tools, and Unfixed gaps, which are not and thus require advanced computational strategies (Fig. 2c). For the Unfixed gaps, the flanking sequence data are fed into the pretrained deep learning model DFillingNet (Fig. 1). The BSCEA algorithm (Table 1) is employed to generate initial, unfiltered sequence predictions to bridge these unresolved gaps.

**Table 1:** Ablation experiments and performance analysis of BSCEA algorithm and DFillingNet architecture on *Micromonas pusilla* dataset

Methods	Network structure	Prediction algorithm	Gap filling num	Gap filling rate(%)
Sealer	–	–	73	51.77%
GapPredict	LTSM	Beam search	77	54.61%
DLGapCloser	DGCNet(CNN+BiLTSM)	Wave-Beam Search	83	58.87%
GapPredict(BSCEA)	LTSM	BSCEA(16)	87	61.70%
Res-2-BiLTSM(BSCEA)	2-BiLTSM+Res	BSCEA(16)	88	62.41%
DL-GapFilling(16)	DFillingNet(CNN+2-BiLTSM+Res)	BSCEA(16)	91	64.54%
DL-GapFilling(32)	DFillingNet(CNN+2-BiLTSM+Res)	BSCEA(32)	94	66.67%
DL-GapFilling(64)	DFillingNet(CNN+2-BiLTSM+Res)	BSCEA(64)	95	67.38%

Stage 3: Hybrid Assembly and Error Correction. The predicted sequences are integrated with the original short-read data in a hybrid assembly process to enhance genomic contiguity and completeness (Fig. 2e). To ensure high fidelity, a dedicated PredictionFilter mechanism (Fig. 2f) performs error correction by comparing the hybrid assembly output against the original scaffold. This critical step validates and refines the predictions, retaining only the most accurate sequences to ensure reliable genome reconstruction.

### PredictionFilter mechanism for predicting sequences

**Figure 3:**
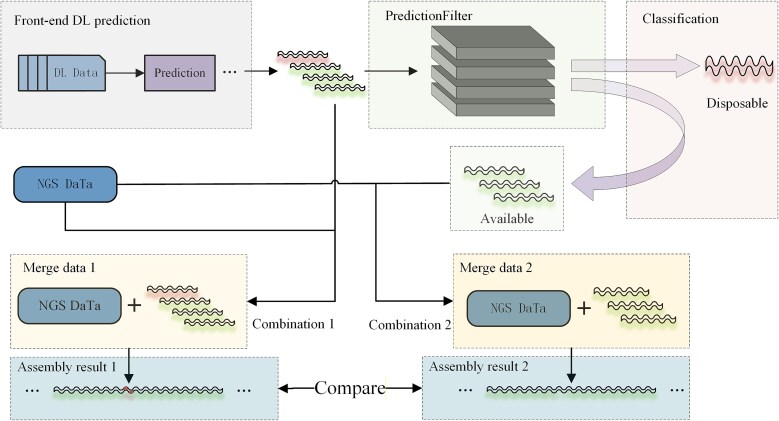
The PredictionFilter is a core screening mechanism designed to evaluate and refine the initial sequences predicted by the DFillingNet model; DFillingNet generates putative gap-filled sequences based on the input flanking regions; however, these raw predictions may contain errors; the PredictionFilter then applies stringent criteria (e.g. alignment score, continuity, and consistency with the original scaffold) to select the most accurate predictions for subsequent hybrid assembly.

The Exonerate tool was used to analyze the alignment files, with alignment rates sorted from 100% to 0%. Higher alignment rates indicate better sequence quality, whereas lower rates reflect poor matches. GapPredict and DLGapCloser directly append predicted sequences to the assembly without evaluating their quality, which may introduce errors. To address this issue, the PredictionFilter mechanism was developed. This mechanism compares the effectiveness of the predicted sequence in filling gaps within homologous gene regions relative to the original assembly. If the prediction improves gap filling, the sequence is classified as available; otherwise, it is discarded. This process ensures that only high-quality predictions are retained, thereby improving the reliability of the assembly (Fig. 3).

After the PredictionFilter mechanism, the predicted sequences are divided into Available sequences and Disposable sequences. Subsequently, the original predicted sequences are combined with the next-generation sequencing data (NGS data) to form Assembly 1; meanwhile, the filtered Available sequences are combined with the NGS data to form Assembly 2. These two combinations enter the assembly step separately, and the assembly results (Assembly results 1 and Assembly results 2) are used to evaluate the effectiveness of the filtering mechanism. The PredictionFilter mechanism enhances assembly quality without altering existing deep learning models. By performing secondary optimization on predicted sequences, it maximizes the potential of current models, providing higher-quality inputs for genome assembly.

## Results

### Gap filling situation

In this experiment, five plant or algal genomes—*Micromonas pusilla*, *Utricularia gibba*, *Eutrema salsugineum*, *Thalictrum thalictroides*, and *Oryza longistaminata*—were selected for analysis. This study used the second-generation Illumina short-read DNA paired-end sequencing data format provided by National Center for Biotechnology Information. DL-GapFilling was evaluated on five different plant or algal datasets using three methods, Sealer [13], GapPredict [45], and DLGapCloser [46]—as baseline methods. The following sections compare the gap-filling performance of these tools across multiple dimensions.

**Figure 4:**
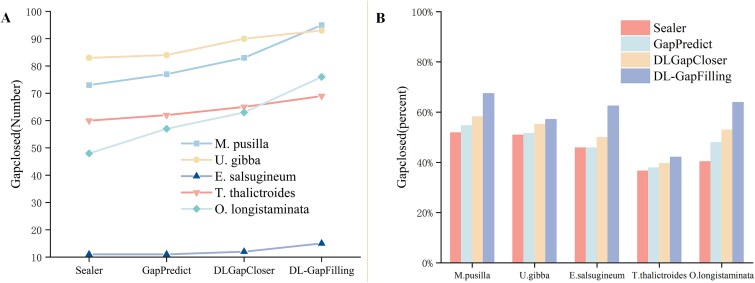
The gap-filling effectiveness of the four methods on five datasets is shown; (a) indicates the number of gaps that can be filled using the four gap-filling methods on five datasets; (b) indicates the proportion of gaps that can be filled using the four methods on each dataset; DL-GapFilling can fill the largest number of gaps on all datasets; from the results, DL-GapFilling performs the best on all datasets and is able to fill the most gaps.

In Fig. 4a, the left panel illustrates the number of gap fills, a key metric for evaluating gap-filling quality. DL-GapFilling significantly enhanced gap filling across all five datasets. Specifically, the number of gap fills improved from 73, 83, and 60 to 95, 93, and 69 for *M. pusilla*, *U. gibba*, and *T. thalictroides*, respectively. For *O. longistaminata*, the number increased from 48 to 76. In Fig. 4b, DL-GapFilling increased the fill rate by 15.6%, 6.1%, and 5.5% for *M. pusilla*, *U. gibba*, and *T. thalictroides*, respectively. For *E. salsugineum*, the fill rate improved by 16.7%, and for *O. longistaminata*, the rate increased from 40.3% to 63.9%, a 23.6% improvement over Sealer and 16% over GapPredict, and 11% over DLGapCloser.

**Figure 5:**
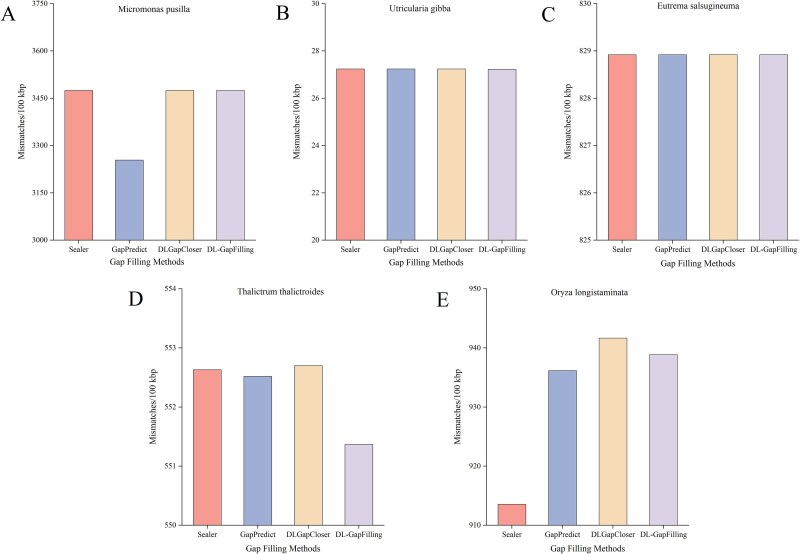
Mismatches in the four assembly methods; DL-GapFilling significantly reduced mismatches in *T. thalictroides*, and in *O. longistaminata* all gap-filling tools that incorporated DL resulted in an increase in mismatches, with a slight decrease in DL-GapFilling.

### Ablation experiments

To evaluate the effectiveness of the DFillingNet model combined with the BSCEA algorithm, ablation experiments were conducted on the *M. pusilla* dataset. The experimental results show that the fill rate of GapPredict is improved to 61.70% after the introduction of BSCEA, which is 7.09% higher than that of traditional Beam Search, demonstrating the advantage of the dynamic contraction–expansion mechanism of BSCEA in optimizing sequence prediction. It especially performs better with an increase in the beam width. The fill rate of DL-GapFilling reaches 64.54%, 66.67%, and 67.38% at beam widths of 16, 32, and 64, respectively (Table 1).

Additionally, the DFillingNet structure significantly improves performance compared to the base LSTM. The fill rate of Res-2-BiLSTM (BSCEA) with the introduction of residual connectivity is improved by 0.71%, showing that residual connectivity helps to mitigate the vanishing gradient problem and enhance feature transfer. Further combining the CNN module with two-layer BiLSTM to construct DFillingNet, the fill rate is improved to 64.54%, which is 2.13% higher than that of Res-2-BiLSTM, verifying the CNN’s ability to extract local features and the advantages of the combination of BiLSTM and residual connectivity in sequence pattern learning. The overall experiment fully proves the superiority of the BSCEA algorithm and DFillingNet structure.

**Figure 6:**
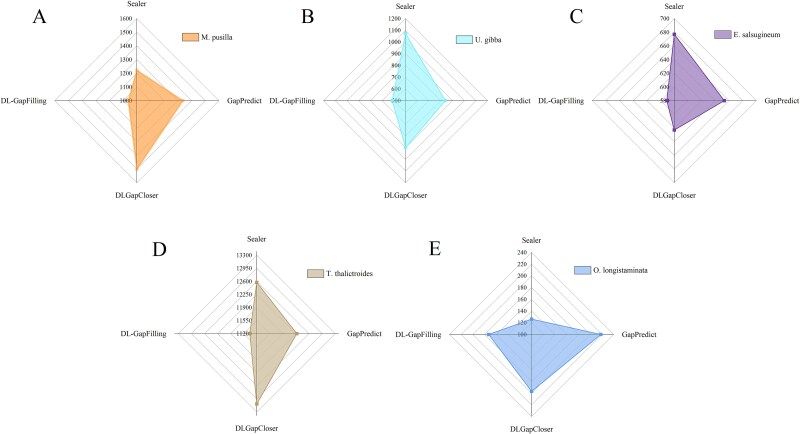
N’s numbers represent the total number of unidentified bases (N) in a genome assembly, which typically arise due to sequencing gaps or unresolved regions; a higher N’s number suggests lower assembly quality, indicating potential sequencing errors or difficulties in assembling repetitive regions.

**Figure 7:**
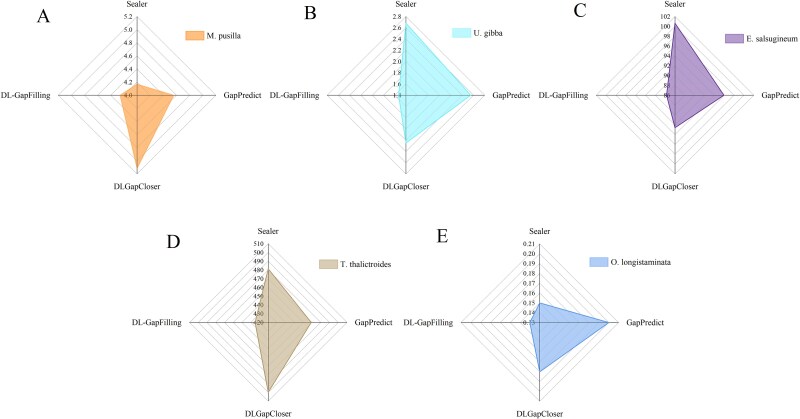
N’s per 100 kb refers to the number of undetermined bases (N) within every 100 000 base pairs (bp) of a genome assembly, serving as an indicator of assembly quality; a lower value suggests fewer unresolved regions and a more complete assembly, reflecting the effectiveness of the gap-filling method; DL-GapFilling outperforms other methods by achieving the lowest N’s per 100 kb across five datasets.

**Figure 8:**
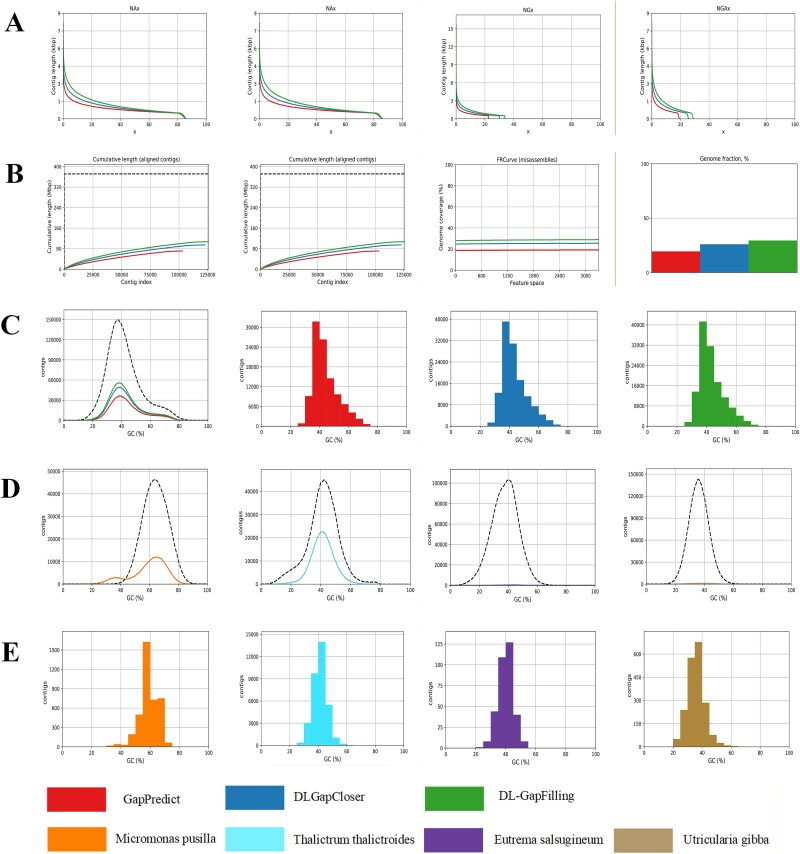
In the *O. longistaminata* dataset, Fig. 8a shows the number of Nx, NAx, NGx, and NGAx for the three deep learning-based assembly methods; Figure 8b shows the cumulative length, cumulative length (aligned contigs), FRCurve (misassemblies), and genome fraction for the three deep learning-based assembly methods; Figure 8c shows the GC content distribution on the *O. longistaminata* dataset; Figure 8d shows the GC content profiles on the *M. pusilla*, *T. thalictroides*, *E. salsugineum*, and *U. gibba* datasets, respectively; the dotted line represents the actual GC content of the reference genome, and the solid line represents the result after assembly by the existing method; Figure 8e visualizes the distribution of GC content in the contigs of these four plant or algal datasets.

### Missing and mismatches in assembly

While no current method can fully resolve all genome assembly gaps, algorithmic optimization continues to enhance assembly accuracy. Scaffolds often contain mismatches, unmapped bases, or numerous N bases in gap regions. Common errors—such as N bases, mismatches, and indels—are evaluated per 100 kb to assess assembly quality. Figure 5 compares mismatch rates across four methods. DL-GapFilling showed improvements for *U. gibba* and *T. thalictroides*, and performed comparably on *E. salsugineum.* For *M. pusilla* and *O. longistaminata*, it slightly increased mismatches but still outperformed other deep learning methods. Indel levels remained consistent across all methods, indicating that DL-GapFilling enhances mismatch correction without introducing new structural errors. With respect to the number of N bases, DL-GapFilling performs slightly worse than Sealer on the *O. longistaminata* dataset but outperforms both GapPredict and DLGapCloser. In contrast, DL-GapFilling surpasses Sealer, GapPredict, and DLGapCloser on the other four datasets, making it the optimal method (Fig. 6). The suboptimal performance on *O. longistaminata* is likely due to its highly repetitive genomic regions, which hinder sequence differentiation and affect deep learning-based methods. Figure 7 shows the average number of mismatches per 100 000 aligned bases (N’s per 100 kb). DL-GapFilling consistently outperforms the other methods across all datasets, achieving results comparable to Sealer on *O. longistaminata*.

### Multi-scale advantages of DL-GapFilling

The assembly quality of several deep learning–based methods was comprehensively evaluated using the QUAST [61] assessment tool, which uses multiple metrics targeting different aspects of genome assembly performance. These metrics include Nx, NAx, NGx, NGAx, GC content, cumulative length (aligned overlapping groups), Feature Response Curve (FRCurve), and genome fraction. Figure 8a illustrates the distribution of Nx, NAx, NGx, and NGAx for three deep learning-based assembly methods, applied to the *O. longistaminata* dataset. Specifically, Nx represents the percentage of assembled bases in the longest contigs, while NAx (with “A” denoting alignment) integrates both the Nx values and the number of misassemblies identified through Plantagora. NGx (Genome Nx) represents the contig length at which x% of the reference genome is covered by contigs of that length or longer, and NGAx further segments contigs into aligned blocks to assess NGx statistics in these blocks. Among the evaluated methods, DL-GapFilling stands out, consistently outperforming both GapPredict and DLGapCloser across all metrics, indicating its superior gap-filling capabilities.

In Fig. 8b, we present additional metrics, including cumulative length, cumulative length (aligned contigs), FRCurve (misassemblies), and genome fraction, calculated for the three deep learning-based assembly methods on the *O. longistaminata* dataset. Cumulative length reflects the total length of all contigs or scaffolds in the assembly, while cumulative length (aligned contigs) specifically accounts for the contigs that can be aligned to the reference sequence. Notably, DL-GapFilling generates longer contigs, thus enhancing the overall assembly. The FRCurve offers a detailed evaluation of the number of misassemblies across different assembly results. In this comparison, DL-GapFilling demonstrates the lowest misassembly rate, confirming its ability to produce more accurate assemblies. Genome fraction measures the proportion of overlap between the assembly and the reference genome, providing insight into the coverage and completeness of the assembly. DL-GapFilling not only exhibits a higher overlap but also aligns more closely with the actual gene structure, further validating its effectiveness in maintaining genomic integrity.

Figure 8c presents the GC content distribution of the assembly results on the *O. longistaminata* dataset, offering an assessment of whether the assemblies preserve the genomic GC characteristics [62]. The horizontal axis represents the GC content percentage, and the vertical axis denotes the overlap group. The first graph provides a general overview of the GC content across all datasets, while the subsequent graphs focus on the GC distribution in the scaffolds generated by each assembly method. It is evident that DL-GapFilling generates results with a GC content distribution that is more consistent with the expected genomic characteristics.

Finally, Fig. 8d visualizes the GC content profiles for datasets from four additional plant or algal species: *M. pusilla*, *T. thalictroides*, *E. salsugineum*, and *U. gibba*. Here, the dotted line represents the actual GC content of the reference genome, and the solid line shows the results after assembly by existing methods. The comparison reveals that current assembly techniques fail to accurately reproduce the GC distribution, further supporting the rationale for incorporating GC content evaluation in the BSCEA. Figure 8e illustrates the distribution of GC content in the overlapping groups across these four plant or algal datasets, providing additional insights into the variation in GC content among different plant or algal genomes [63].

After the comprehensive analysis, it is evident that DL-GapFilling consistently outperforms existing assembly models across several critical evaluation metrics, including Gapclosed Number, Gapclosed (percent), mismatches per 100 kb, N’s numbers, N’s per 100 kb, and GC content. Specifically, DL-GapFilling surpasses both traditional assembly tools and those integrated with deep learning, demonstrating its clear superiority, particularly on plant or algal datasets. The method excels not only in improving the number of gaps closed but also in achieving a higher gap-filling percentage, which is crucial for enhancing the completeness of the assembled genomes. Additionally, DL-GapFilling shows a significant reduction in mismatches, indicating its ability to produce more accurate sequences with fewer errors. Furthermore, the GC content profile generated by DL-GapFilling aligns more closely with the expected values, showcasing its effectiveness in maintaining genomic integrity during assembly. This comprehensive performance across multiple quality metrics highlights DL-GapFilling as an optimal approach for genome assembly, particularly in plant or algal genomes, where traditional and current deep learning-based methods fall short.

## Discussion

Our DL-GapFilling framework introduces a novel paradigm for complex gap resolution by strategically integrating deep learning. Instead of a computationally prohibitive end-to-end deep learning approach, we target deep learning specifically to gaps that resist conventional algorithms and complement it with a predictive filtering mechanism. This efficient hybrid strategy significantly improves assembly quality and pioneers a practical path for genome assembly powered by Artificial Intelligence (AI).

The remarkable success of DL-GapFilling lies in its innovative approach and key technical advancements. First, the hybrid network architecture combining residual networks and BiLSTM networks harnesses the advantages of contextual modeling, which is particularly effective for capturing the complex relationships present in genomic sequences. Second, the introduction of the BSCEA has proven to be instrumental in optimizing the sequence expansion process, significantly improving prediction efficiency while simultaneously reducing errors. The BSCEA algorithm’s ability to adaptively adjust gap expansion helps achieve more accurate gap filling and contributes to a more complete genome reconstruction. Third, the PredictionFilter mechanism further enhances the model’s performance by refining predicted sequences, ensuring that only high-quality predictions are incorporated into the final assembly, without the need for model retraining. These innovative approaches—combined with a carefully designed architecture—are what set DL-GapFilling apart from existing methods.

Despite the promising results, DL-GapFilling has several limitations that warrant further investigation. First, its performance has been validated primarily on plant or algal genomes, such as *O. longistaminata*, leaving its generalizability to animal and microbial genomes untested. Second, while the method addresses gaps in second-generation sequencing data, third-generation platforms introduce distinct challenges, which may demand specialized gap-filling strategies. Future work will focus on scaling DL-GapFilling to ultra-large and non-model genomes, as well as developing tailored approaches for third-generation data to improve assembly continuity and reference genome quality.

**Key Points**

- We construct a DFillingNet model and propose a BSCEA algorithm based on A^*^ and BS, which for the first time considers the characteristics of the sequences themselves to perform a beamwidth dynamic adjustment strategy to improve the prediction accuracy.
- For the first time, we introduce a filtering mechanism for the predicted sequences generated by deep learning and successfully improve the gap filling quality through the filtered predicted sequences.
- We have constructed a new dataset and evaluation standard covering a wide range of organisms, which provides a reliable reference for gap filling in a wide range of genomes.

## Data Availability

The DL-GapFilling model, toolkit and dataset are available at https:/github.com/zihaowangcs/DL-GapFilling.
